# Supplementary material for: Direct contribution of skeletal muscle mesenchymal progenitors to bone repair
Source: Nat Commun. 2021 May 17;12:2860. doi: 10.1038/s41467-021-22842-5 (PMC8128920; doi:10.1038/s41467-021-22842-5)
Supplement: Supplementary file 1 — Supplementary Information [file 41467_2021_22842_MOESM1_ESM.pdf]

Direct contribution of skeletal muscle mesenchymal progenitors to bone repair.

Anais Julien, Anuya Kanagalingam, Ester Martinez-Sarra, Jérome Megret, Marine Luka, Mickaël Ménager, Frédéric Relaix & Céline Colnot

Supplementary information

Supplementary Figure 1

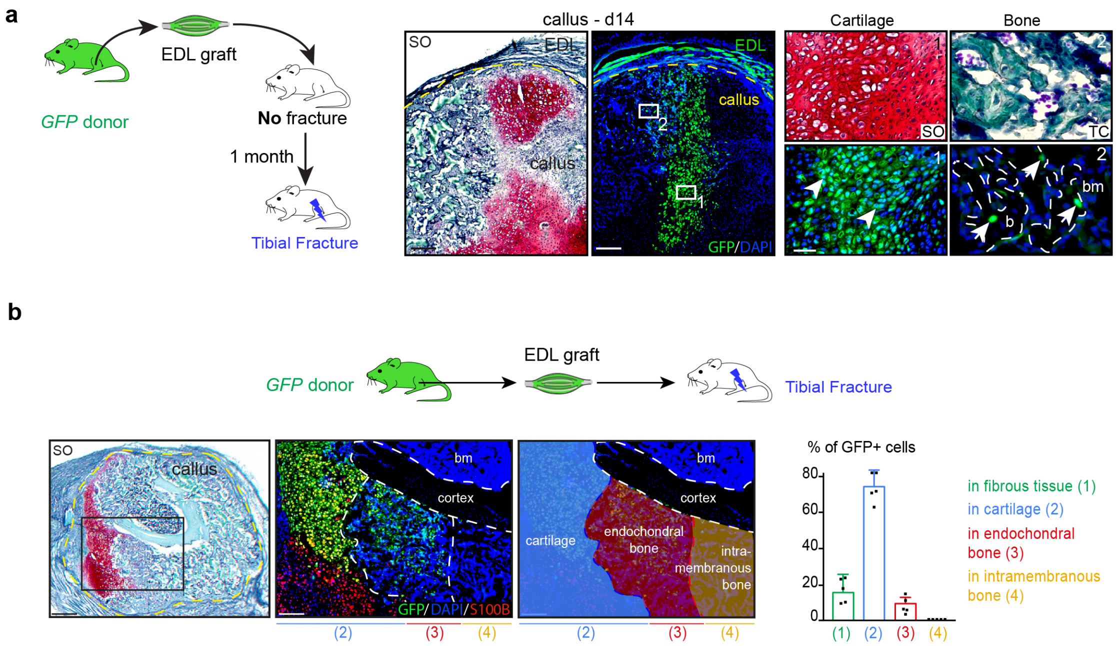

Supplementary Figure 1: Skeletal muscle directly contributes to cartilage and bone during bone repair, related to Figure 1

a, Experimental design of non-stabilized tibial fracture induced one month after GFP-EDL skeletal muscle graft transplantation in wild type hosts. Callus sections of GFP-EDL skeletal muscle graft next to the fracture callus (delimited by a yellow dotted line) at d14 post-fracture stained with safranin'O (SO, left) and counterstained with DAPI (right). High magnification of cartilage (box 1) and bone (box 2, b, white dotted line) containing GFP+ EDL skeletal muscle-derived chondrocytes (white arrowhead) and osteocytes respectively (white arrow). **b,** Experimental design of GFP-EDL skeletal muscle graft at the time of fracture of wild type hosts. Representative image of calluses at d14 post-fracture stained with SO and adjacent section immunostained with S100b antibody (in red) and counterstained with DAPI. Image on the right shows the delimitation of cartilage, endochondral bone and intramembranous bone areas used for quantification. Quantification of percentage of GFP+ cells in fibrous tissue (1, green), cartilage (2, blue), endochondral bone (3, red) and intramembranous bone (4, orange), normalized on the total number of GFP+ cells within the callus. n=5, bm: bone-marrow. DAPI in blue, GFP in green. Scale bar: low magnification: 1mm, high magnification: 50µm. Representative images of 3 distinct samples. All data represent mean ± SD. Images are representative of 2 independent experiments.

Supplementary Figure 2

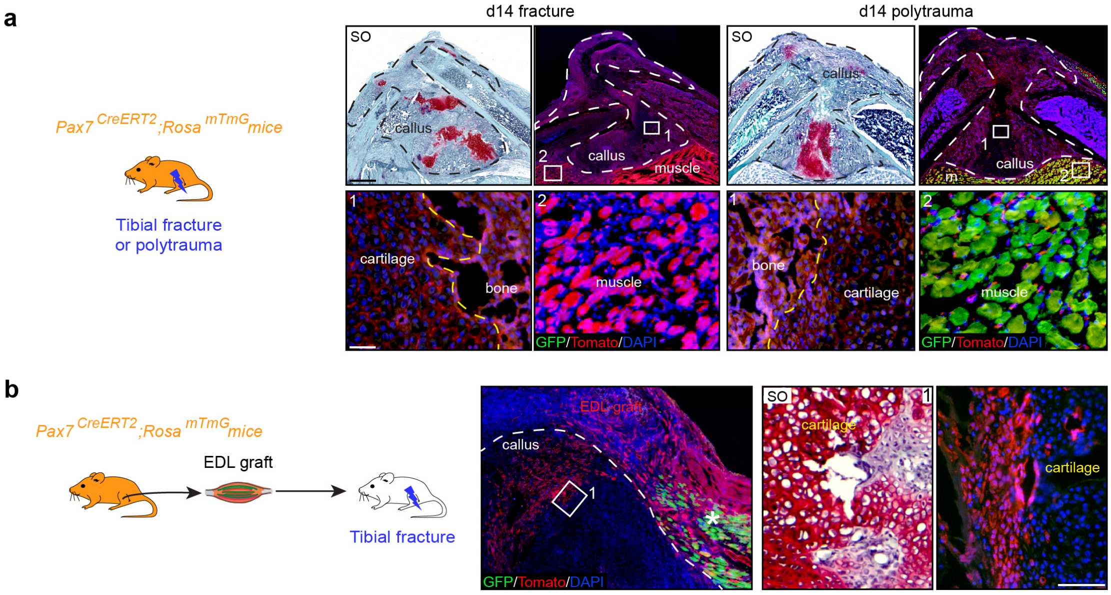

Supplementary Figure 2: Myogenic lineage does not contribute to bone repair, related to Figure 1 **a**, Experimental design. Longitudinal callus section at d14 after non-stabilized tibial fracture (left) or polytrauma (right) in tamoxifen-induced *Pax7*^{CreERT2}*;Rosa*^{mTmG} mice. Top: Representative images stained with safranin'O (SO) (callus delimited by a black dotted line) and adjacent sections counterstained with DAPI. Bottom: High magnification of callus (box1) and muscle (box2) shows no GFP signal in cartilage or bone and GFP+ new myofibers next to the callus in mice after polytrauma. **b**, Experimental design of EDL skeletal muscle grafts from *Pax7*^{CreERT2}*;Rosa*^{mTmG} mice transplanted adjacent to the fractured tibia in wild-type hosts. Longitudinal callus section counterstained with DAPI showing EDL-derived cells (Tomato+) within the callus (delimited by a white dotted line) and regenerating myofibers within the graft (GFP+, asterisk). Enlarged view of boxed area 1 in cartilage stained with SO and counterstained with DAPI shows only Tomato+ chondrocytes and absence of GFP+ chondrocytes. DAPI in blue, GFP in green, Tomato in red. Scale bar: low magnification: 1mm, high magnification: 50µm. Representative images of 3 distinct samples.

Supplementary Figure 3

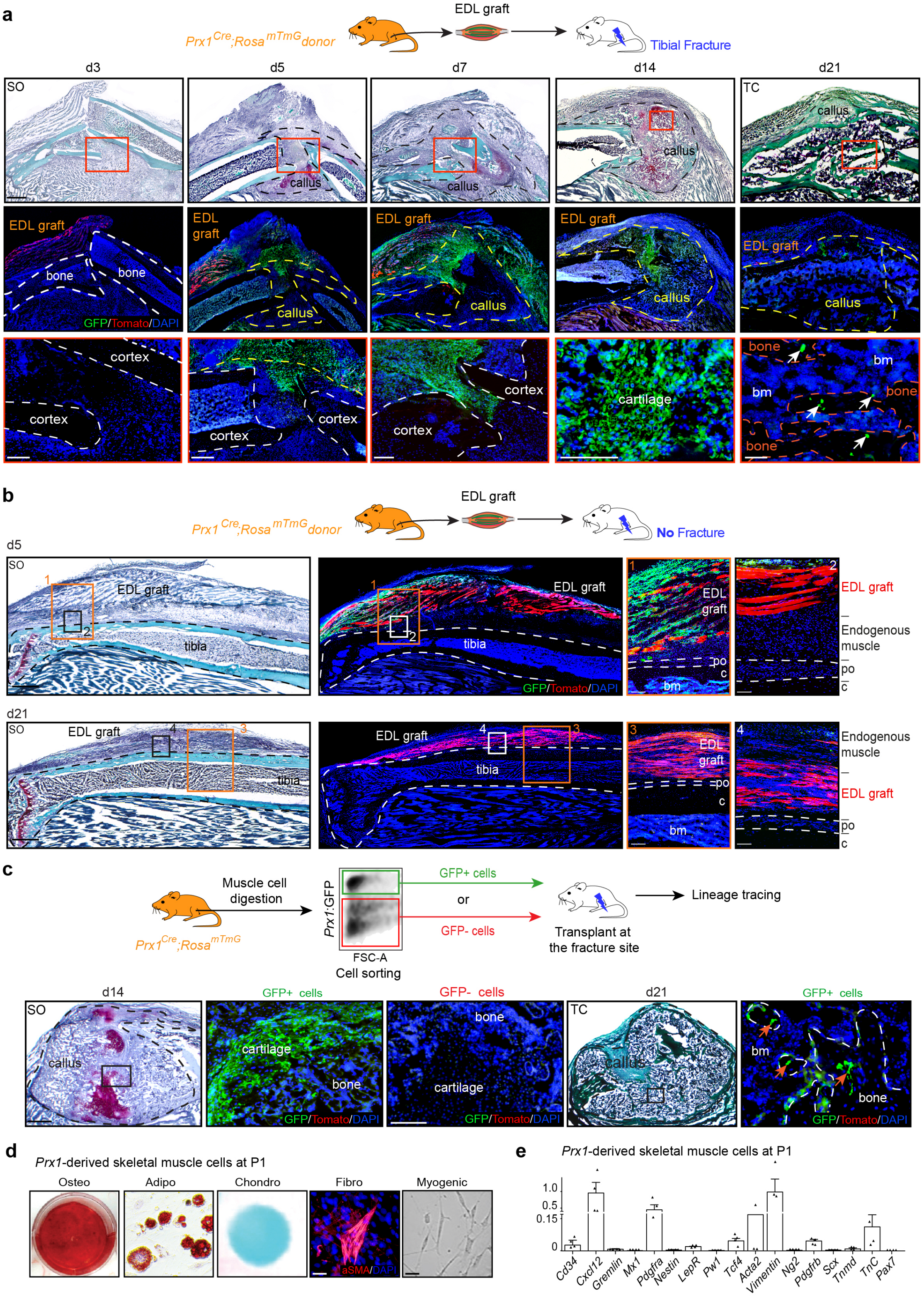

Supplementary Figure 3: Lineage analyses of skeletal muscle-derived cells contributing to bone repair, related to Figure 1

a, Experimental design of *Prx1*^{Cre}*;Rosa*^{mTmG} EDL skeletal muscle graft transplanted at the fracture site of wild type hosts. Top and middle: Representative sections of fracture calluses at days 3, 5, 7, 14 and

21 post non-stabilized tibial fracture stained by safranin'O (SO) or Masson's trichrome (TC) (callus delimited by a black dotted line) and adjacent sections counterstained with DAPI (callus delimited by a yellow dotted line). Bottom: high magnification–of red boxed areas showing the migration of graft derived cells into the callus from d5 (bone cortices delimited by a white dotted line), differentiation into chondrocytes between d7 and d14 and into osteocytes at d21 post-fracture (white arrows, new bone delimited by an orange dotted line). Scale bar: SO/TC: 1mm, high magnification d3, d5, d7: 250 µm, d14: 50µm and d21: 25 µm. **b**, Experimental design of *Prx1^{Cre};Rosa^{mTmG}* EDL skeletal muscle graft transplanted next to unfractured tibia of wild-type hosts. Longitudinal sections of hindlimb at d5 and d21 post-transplantation stained with SO and adjacent sections counterstained with DAPI. High magnifications (boxed areas 1-4) show GFP+ cells within grafted EDL skeletal muscle at d5 post-transplantation, but no GFP+ cells in adjacent endogenous muscles, periosteum (po) or bone marrow (bm) at d5 and d21 post-transplantation. Scale bar: SO/TC: 1mm, high magnification, boxed areas 1 and 3: 100 µm, boxed areas 2 and 4: 50µm. **c**, Experimental design of skeletal muscle cells grafted at the fracture site of wild type hosts. Skeletal muscle cells were isolated from skeletal muscle of *Prx1^{Cre};Rosa^{mTmG}* mice and transplanted at the fracture site of wild type mice following cell sorting. Callus sections stained with SO at d14 post-fracture and with TC at d21 post-fracture and visualization of Prx1-derived (GFP+) and non Prx1-derived (Tomato+) skeletal muscle cells on adjacent sections counterstained with DAPI. High magnification of cartilage and bone (b, white dotted line) areas. Scale bar: SO/TC: 1mm, high magnification for cartilage 50 µm, and for bone 25µm. po: periosteum, bm: bone marrow, c: cortex. Representative images of at least 3 distinct samples. **d**, Osteogenic (alizarin red staining), adipogenic (oil red o staining), chondrogenic (alcian blue staining), fibrogenic (aSMA immunocytochemistry) and myogenic (phase contrast) *in vitro* differentiation of Prx1-derived skeletal muscle cells. Representative images from 3 independent samples. Scale bar: 10µm**.** **e**, RT-qPCR analysis on sorted Prx1-derived skeletal muscle cells from *Prx1^{Cre};Rosa^{YFP}* mice at P1. n=4 animals per group. DAPI in blue, GFP in green, Tomato in red. All data represent mean ± SD. Images are representative to 2 independent experiments.

Supplementary Figure 4

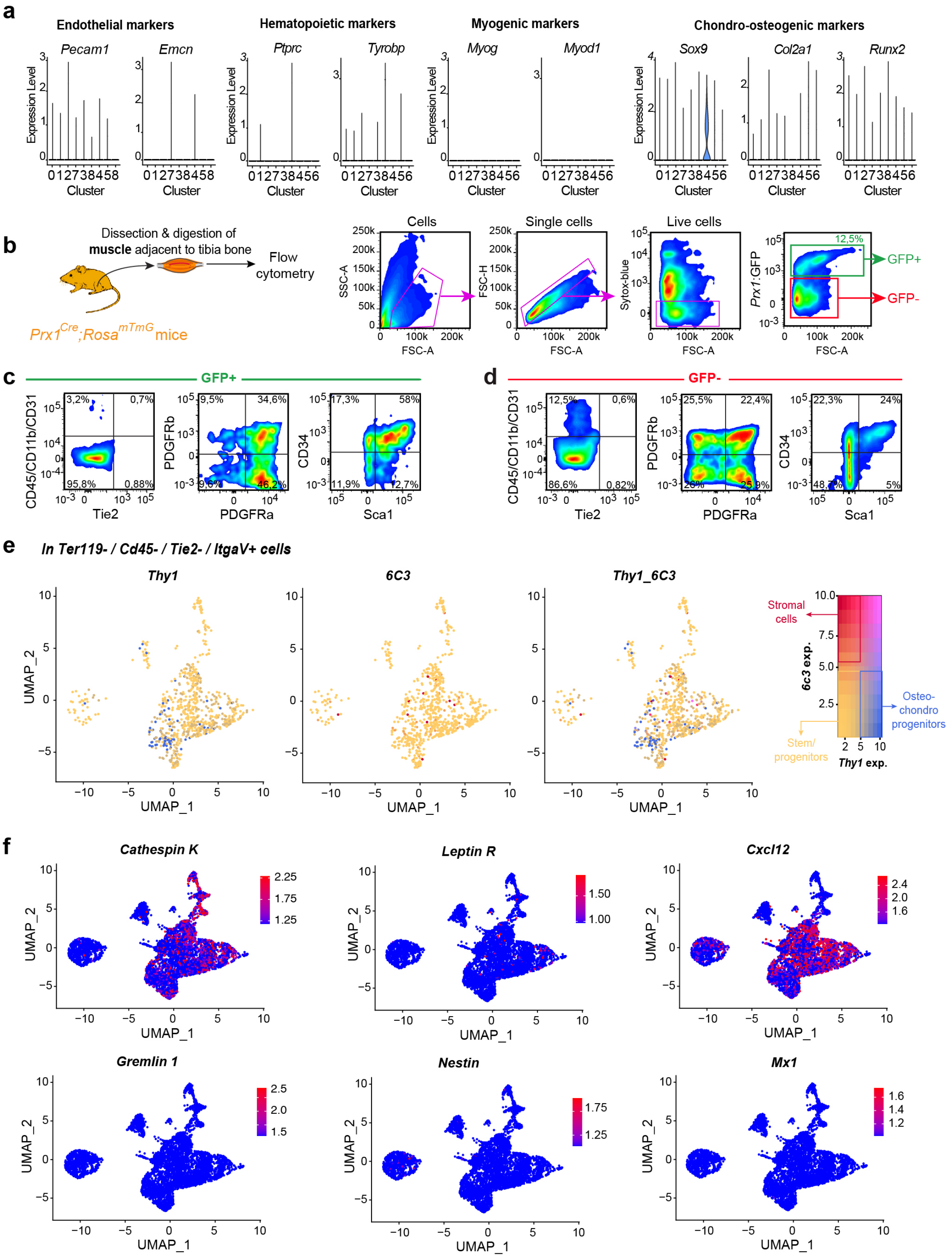

Supplementary Figure 4: Single-cell RNAseq analyses of skeletal muscle mesenchymal progenitors, related to Figure 2

a, Expression of endothelial, hematopoietic, myogenic and chondro-osteogenic markers at d0 in scRNA-seq. **b**, Left, Experimental design of flow cytometry analysis. Right, Schematic representation of gating strategy of skeletal muscle cells used for flow cytometry analysis. **c**, **d**, FACS plots displaying the distribution of PDGFRβ, CD29, PDGFRα, Sca1 and CD34 cells in the GFP+/CD45-CD11b-CD31-

population (**c**) and in the GFP-/CD45-CD11b-CD31- double negative population (**d**). GFP- and GFP+ populations are gated as showed in panel b. Values represent the average of 3 independent experiments. **e**, Feature plot expression of *6C3* and I markers in *Ter119-/Cd45-/Tie2-/ItgaV+* sub-population. According to Chan et al[1], *6C3/Thy1* double negative cells correspond to stem/progenitor cells (orange dots), *6C3+/Thy1-* correspond to stromal cells (red dots) and *6C3-/Thy1+* correspond to osteochondroprogenitors (blue dots). **f**, Feature plots expression of genes described in the literature as skeletal stem cell markers[2–7].

Supplementary Figure 5

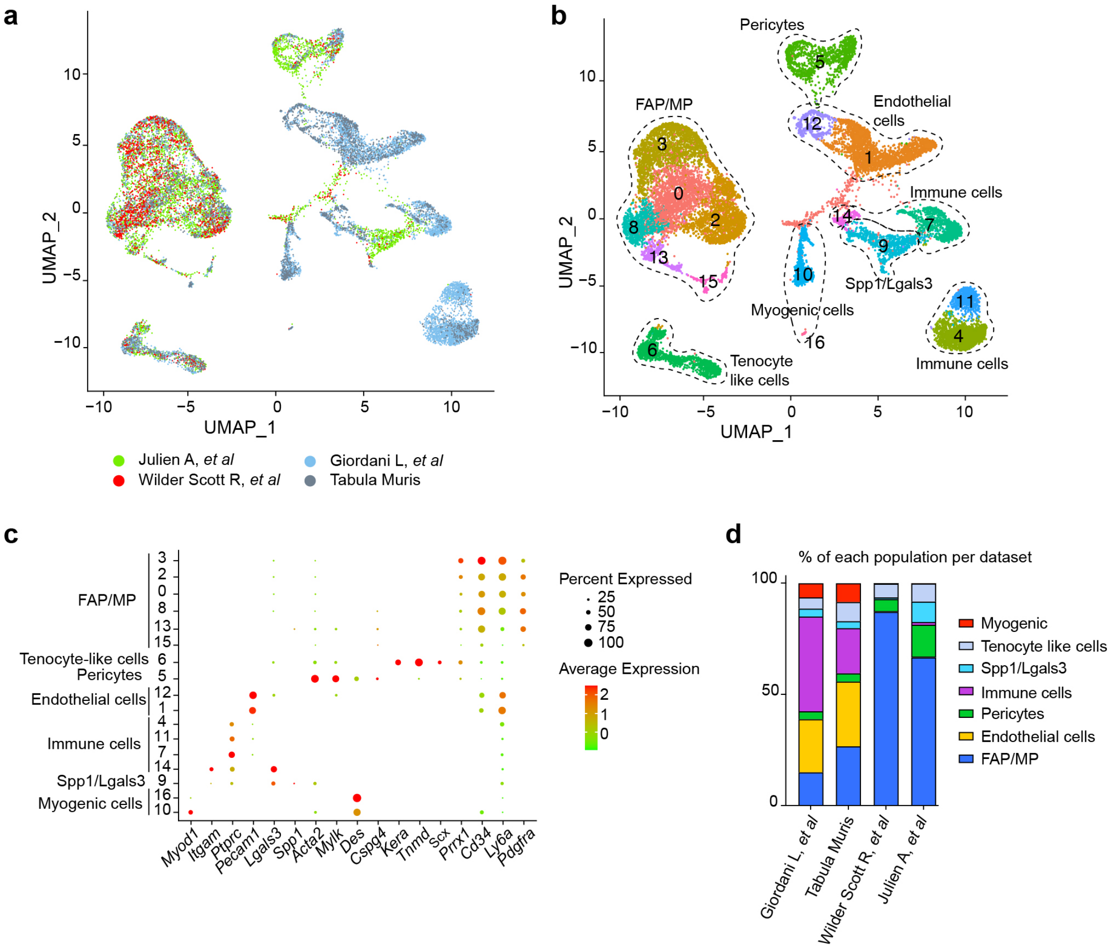

Supplementary Figure 5: Comparative single-cell RNAseq analyses of Prx1-derived skeletal muscle cells and previously reported skeletal muscle datasets, related to Figure 2.

a, UMAP projection of the 4 datasets integrated: whole mononucleated cells from Tabula Muris consortium[8] (grey dots), Giordani L. et al[9] (blue dots), *Hic1*^{CreERT}*;Rosa*^{dtTom} sorted skeletal muscle cells from Wilder Scott R.[10] (red dots) and *Prx1*^{Cre}*;Rosa*^{mTmG} sorted skeletal muscle cells (green dots). **b**, Unsupervised clusterization of the 4 datasets integrated results into 17 clusters. Cell populations are delimited by a black dotted line. **c**, Expression specific markers defining 7 populations within skeletal muscle. **d**, Percentage of each sub-population per dataset.

Supplementary Figure 6

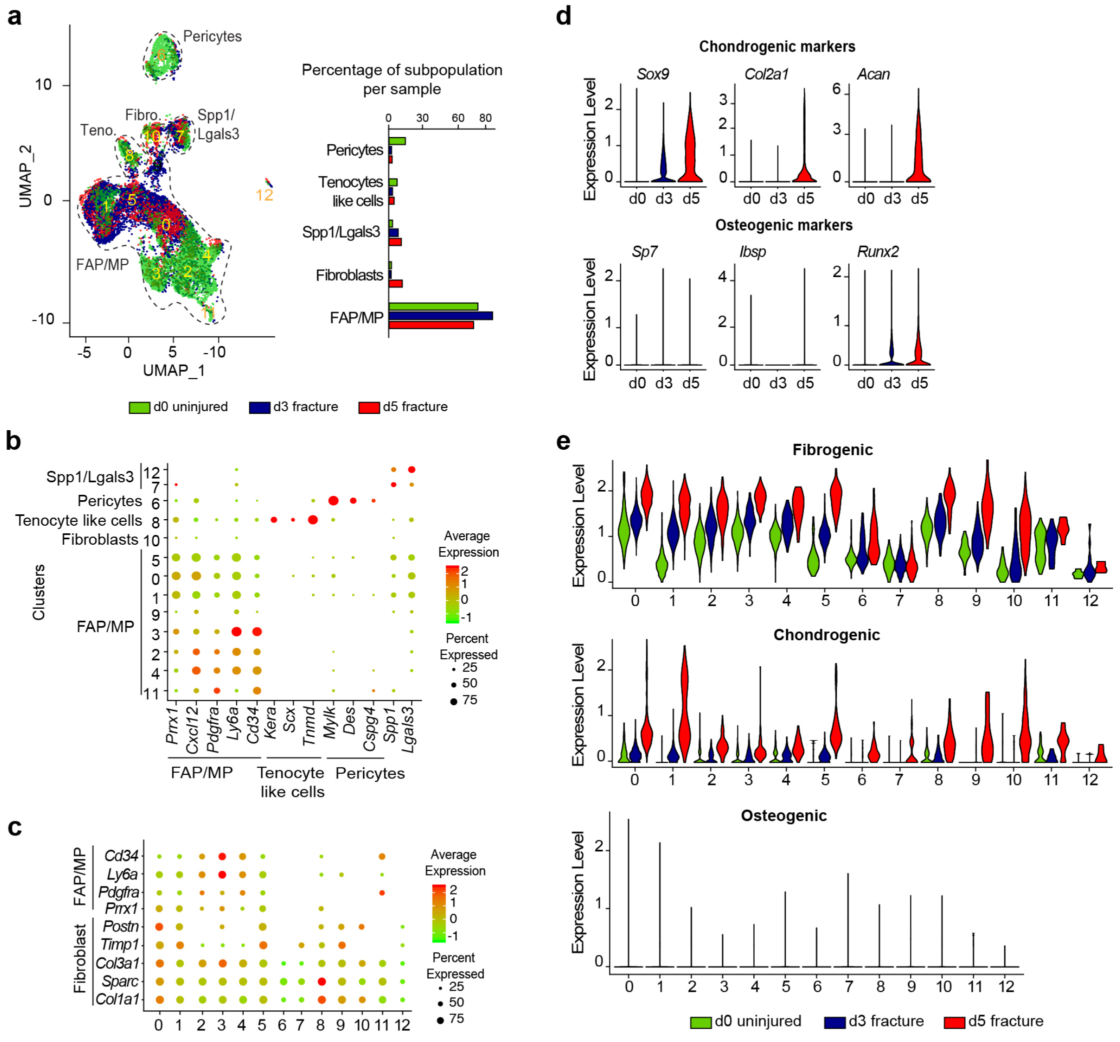

Supplementary Figure 6: Single-cell RNAseq analysis of skeletal muscle mesenchymal progenitors in response to bone fracture, related to Figure 3

a, Left: UMAP visualization of integrated data from d0, d3 and d5 Prx1-derived skeletal muscle cells. Right: Percentage of subpopulation per sample in combined analysis of d0, d3 and d5 post-fracture samples. **b**, Dotplot of indicated genes expression identifying FAP/MP, tenocyte-like cells, pericytes and Spp1/Lgals3 cell populations. **c**, Dotplot of FAP/MP and fibroblast genes expression. **d**, Pseudobulk expression of chondrogenic and osteogenic markers in d0, d3 and d5 post-fracture samples. **e**, Mesenchymal, fibrogenic, chondrogenic and osteogenic lineage score in d0, d3 and d5 post-fracture samples per cluster. Teno.: tenocyte like cells, Fibro.: fibroblasts.

Supplementary Figure 7

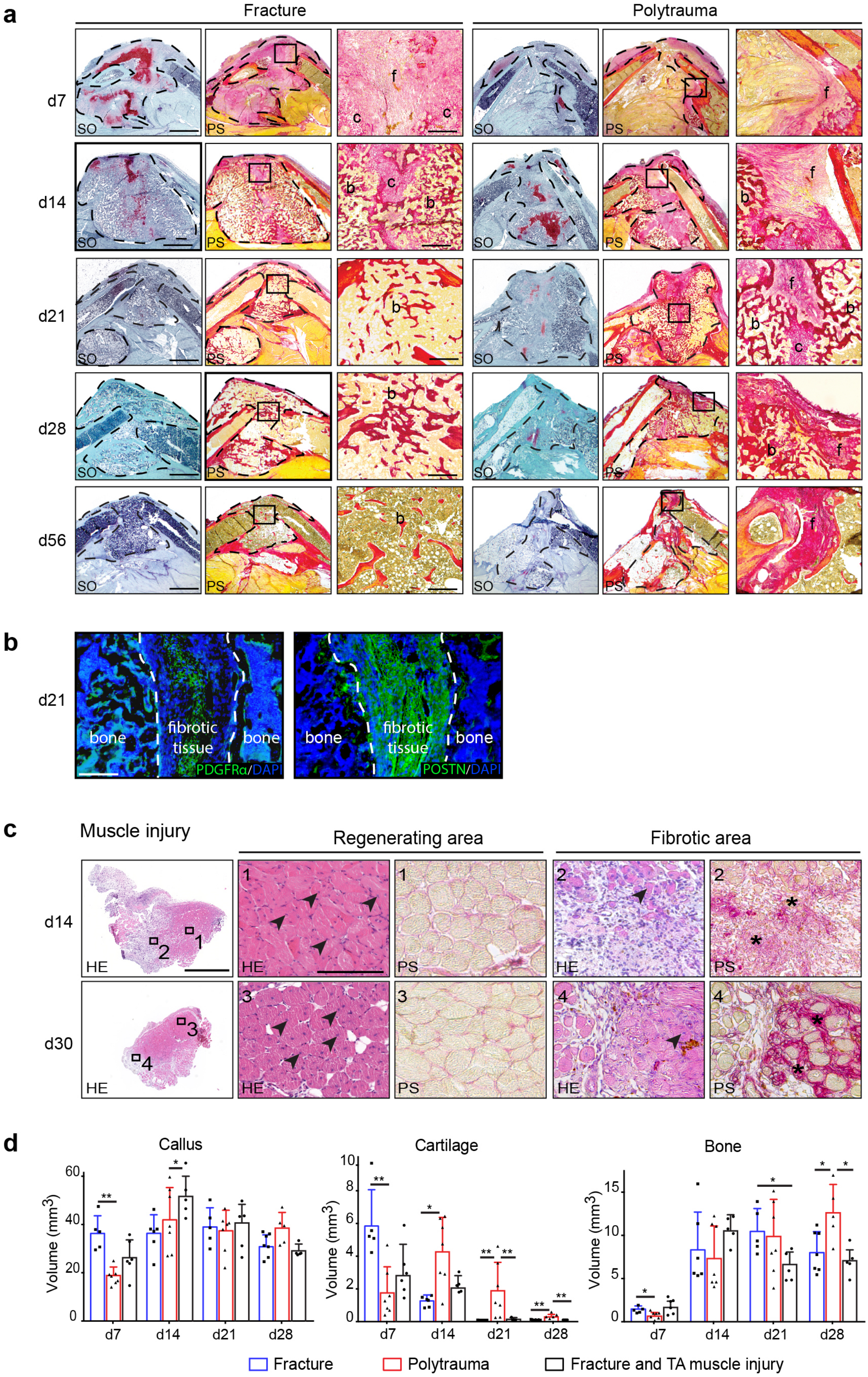

Supplementary Figure 7: Impact of skeletal muscle injury on bone repair, related to Figure 4

a, Representative sections of non-stabilized tibial fracture calluses stained with safranin'o (SO) and picro-sirius (PS) at days 7, 14, 21, 28 and 56 post-fracture (left panels) and post-polytrauma (right panels) (callus outlined with a black dotted line, high magnifications of boxed areas). f: fibrosis, c: cartilage, b: bone, Scale bar=1mm, box areas =50μm. **b**, Immunostaining of PDGFRα (green, left) and Periostin (POSTN, green, right) in fibrosis (delimited by a white dotted line) of wild-type callus. Scale bar: 50μm. **c**, Tibialis anterior (TA) muscle sections at 14 and 30 days post-skeletal muscle injury alone (upper and lower panel respectively) stained with Hematoxylin and Eosin (HE) and PS. High magnifications show centronucleated myofibers (arrowheads) in the regenerating area (boxes 1,3) and centronucleated myofibers surrounded by fibrous tissue (asterisks) in the fibrotic area (boxes 2,4). Representative images of 3 distinct experiments. Scale bar=1mm, box areas =50μm. **d**, Histomorphometric quantification of callus, cartilage and bone volumes in fractures without muscle injury, with total muscle injury (as shown in Figure 1) or with TA muscle injury only at days 7, 14, 21 and 28 post-fracture. d7 n=6, d14 n=5, d21 n=5, d28 n=5. Statistical analyses were performed following two-sided Mann-Whitney test and exact p-values are indicated in the graph. All data represent mean ± SD.

Supplementary Figure 8

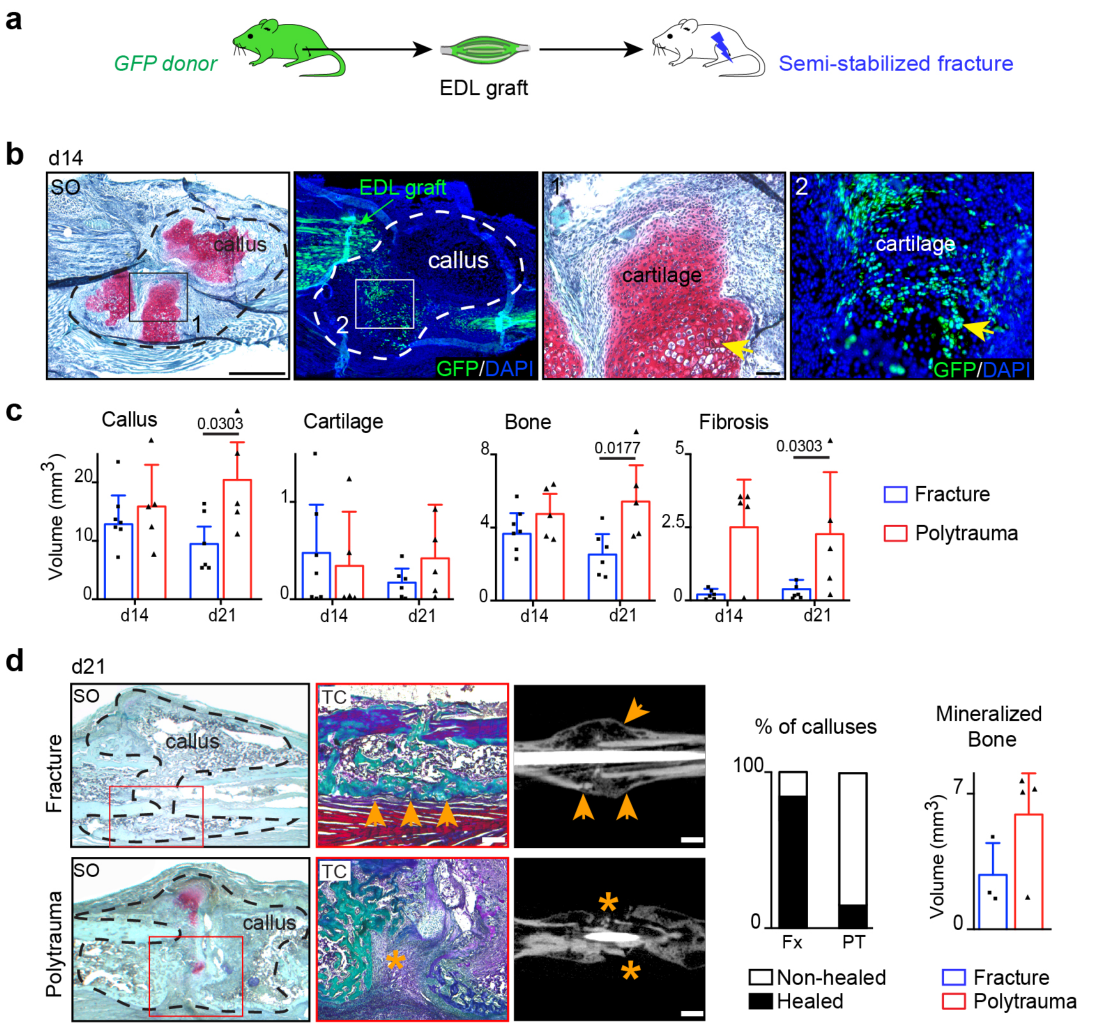

Supplementary Figure 8: Skeletal muscle injury impairs bone regeneration in a semi-stabilized fracture model.

a, Experimental design of EDL skeletal muscle graft from *GFP*-mice transplanted at the fracture site of semi-stabilized tibial fracture of wild type hosts. **b**, Longitudinal callus sections stained with safranin'O (SO) and adjacent section counterstained with DAPI at d14 post-fracture (callus delimited by dotted line). High magnification of boxed areas show GFP+ skeletal muscle-derived cells within cartilage (yellow arrow). DAPI in blue, GFP in green. **c**, Histomorphometric analyses of callus, cartilage and bone volumes at d14 and d21 post-fracture or post-polytrauma (d14 fracture n=7, d14 polytrauma n=5, d21 fracture n=6, d21 polytrauma n=5. **d**, Longitudinal callus sections stained with SO, Masson's trichrome (TC) and micro-CT images at d14 and d21 post-fracture (top) or post-polytrauma (bottom). High magnification of red boxed area show defect in bone bridging after polytrauma (asterix) compared to fracture alone (orange arrowhead). Percentage of healed and non-healed calluses (fracture n=7, polytrauma n=7) (Fx: fracture, PT: polytrauma). Quantification of volume of mineralized bone based on micro-CT images (fracture n=3, polytrauma n=4). Statistical analyses were performed following two-sided Mann-Whitney test and exact p-values are indicated in the graph. All data represent mean ± SD.

Supplementary Figure 9

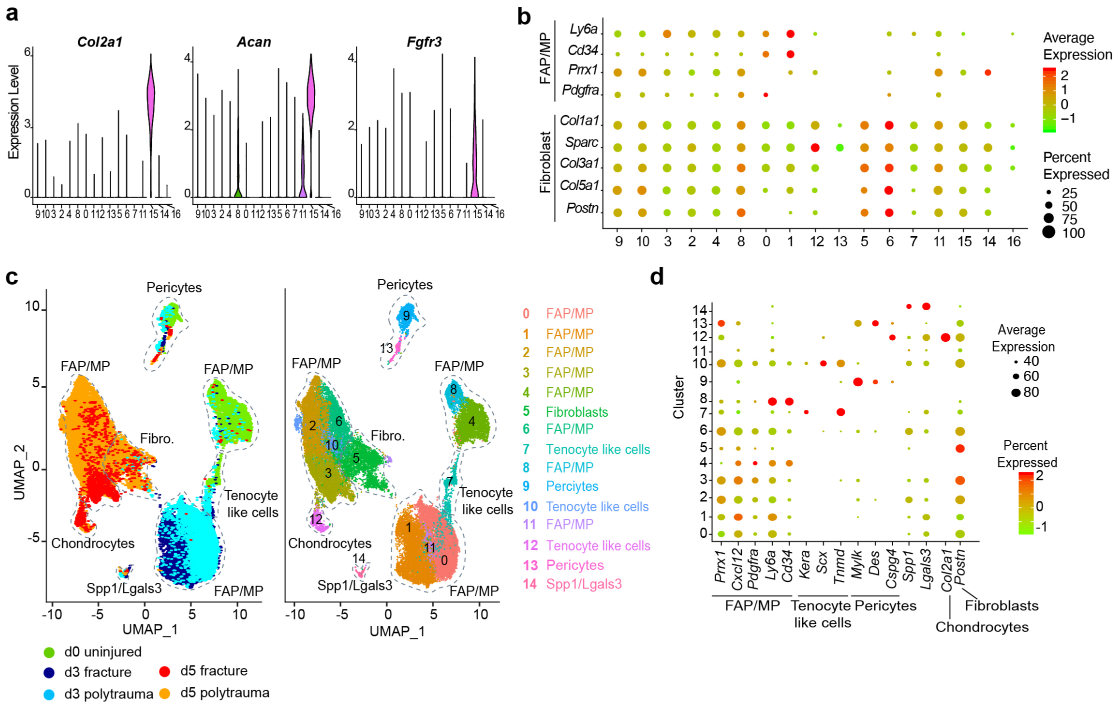

Supplementary Figure 9: Single-cell RNAseq analysis of skeletal muscle mesenchymal progenitors in response to fracture and polytrauma, related to Figure 5

a, Expression of chondrogenic markers among clusters. **b**, Dotplot of fibroblast and mesenchymal markers among clusters in combined analysis of d0, d3 and d5 post-fracture and post-polytrauma samples. **c**, UMAP projection of sample aggregate after cell cycle regression. **d**, Expression of markers identifying clusters in sample aggregate after cell cycle regression.

Supplementary Figure 10

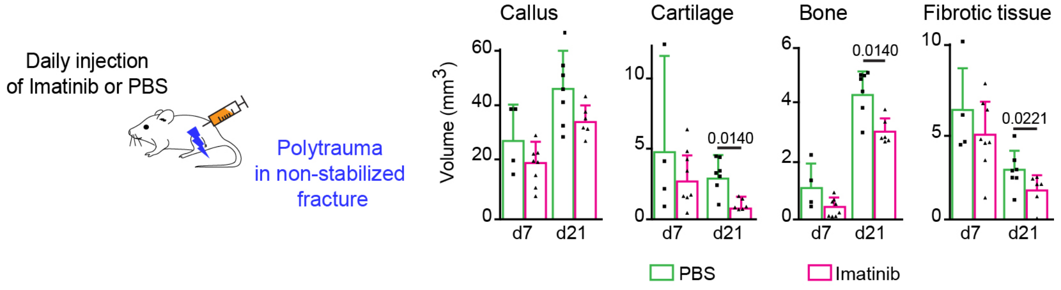

Supplementary Figure 10: Imatinib treatment decreases fibrotic tissue accumulation in non-stabilized fracture model after polytrauma, related to Figure 7.

Daily injection of Imatinib® (50mg/kg/day) or vehicle (PBS) in mice with polytrauma. Histomorphometric analyses of total callus, cartilage, bone and fibrosis volumes of Imatinib-treated and PBS-treated mice at days 7 or 21 post-polytrauma (d7 PBS-treated n=4, d7 Imatinib-treated n=8, d21 PBS-treated n=7, d21 Imatinib-treated n=6). Statistical analyses were performed following two-sided Mann-Whitney test and exact p-values are indicated in the graph. All data represent mean ± SD.

Lineage	Genes used
Fibrogenic	GO list number 0043062
Mesenchymal	"Cd34", "Cxcl12", "Prrx1", "Ly6a", "Pdgfra", "Eng"
Osteogenic	"Alpl", "Sp7", "Ibsp", "Runx2"
Chondrogenic	GO list number 0051216
Cell death	"Casp3", "Casp7", "Bax", "Bak1", "Apaf1", "Bid", "Bcl2", "Mcl1"

Supplementary information, Table 1: Markers used in lineage analysis, related to Figures 2 and 4

Name of signalling pathways in Enrich R	Adjusted P-value	Genes	Linked with chondrogenesis	References
AP-1 transcription factor network	5,01901E-08	*Egr1;Jun;Il6;Maf; Dusp1;Fos;Cyr61;Atf3*	YES	[11,12]
ATF2 transcription factor network	1,26316E-08	*Pdgfra;Jun;Il6;Dusp1; Mmp2;Ddit3;Fos;Atf3*	YES	[13–15]
Diabetes pathways	8,73293E-06	*Igfbp5;Igfbp4;Ddit3; Mmp2;Igfbp6;Igf1; Klf4;Atf3*	NO	
ERBB1 downstream pathway	0,000142914	*Egr1;Zfp36;Jun; Dusp1;Pik3r1;Fos*	YES	[16,17]
Insulin-like growth factor (IGF) activity regulation by insulin-like growth factor binding proteins (IGFBPs)	1,2351E-07	*Igfbp5;Igfbp4;Mmp2; Igf1;Igfbp6*	YES	[18–20]
Nuclear beta-catenin signaling and target gene transcription regulation	0,000329148	*Jun;Mmp2;Id2;Klf4; Cyr61*	YES	[21–23]
Pathways in cancer	0,0007548	*Fzd1;Pdgfra;Fgf7;Jun; Il6;Mmp2;Pik3r1;Igf1; Fos*	NO	
RAGE pathway	8,42527E-05	*Il6;Ace;Id2;Igf1;Icam1*	YES	[24]
Regular glucocorticoid receptor pathway	3,3959E-05	*Egr1;Nr4a1;Jun;Il6; Fos;Icam1*	YES	[25,26]
regulation of canonical Wnt signaling pathway (GO:0060828)	0,000206461	*Col1a1;Sfrp4;Ddx3x;Igfbp4;Ddit3;Igfbp6; Uba52;Dkk2*	YES	[27,28]
Signaling by PDGF	0,00030721	*Col1a1;Pdgfra;Nr4a1; Col3a1;Col2a1;Pik3r1*	NO	

Supplementary information, Table 2: Signaling pathways linked with chondrogenic differentiation, related to Figure 5. Top: Enrich R results of GO enrichment analysis, Statistical analyses were performed following Fischer exact test. Bottom: References used to analyze Enrich R results.

Gene	Primer sequence
Cd34 forward<br>Cd34 reverse	AAGGCTGGGTGAAGACCCTTA<br>TGAATGGCCGTTTCTGGAAGT
Cxcl12 forward<br>Cxcl12 reverse	GAGCCAACGTCAAGCATCTG<br>CGGGTCAATGCACACTTGTC
Mx1 forward<br>Mx1 reverse	GACCATAGGGGTCTTGACCAA<br>AGACTTGCTCTTTCTGAAAAGCC
Gremlin1 forward<br>Gremlin1 reverse	AAGCGAGATTGGTGCAAAACT<br>GAAGCGGTTGATGATAGTGCG
Pdgfra forward<br>Pdgfra reverse	AGAGTTACACGTTTGAGCTGTC<br>GTCCCTCCACGGTACTCCT
Nestin forward<br>Nestin reverse	TCCCTTAGTCTGGAAGTGGCTA<br>GGTGTCTGCAAGCGAGAGTT
Leptin Receptor forward<br>Leptin Receptor reverse	ATGTGCCCTTCCGATATACAACC<br>CGTGTCATCCACTAATCTTCTGG
PW1 forward<br>PW1 reverse	TCATGCACACTAGGGAGAACC<br>GGCAGCACTCCTACTGAAGG
Tcf4 forward<br>Tcf4 reverse	CGAAAAGTTCCTCCGGGTTTG<br>CGTAGCCGGGCTGATTCAT
Acta2 forward<br>Acta2 reverse	GTCCCAGACATCAGGGAGTAA<br>TCGGATACTTCAGCGTCAGGA
Vimentin forward<br>Vimentin reverse	CTGCTTCAAGACTCGGTGGAC<br>ATCTCCTCCTCGTACAGGTCG
NG2 forward<br>NG2 reverse	GGGCTGTGCTGTCTGTTGA<br>TGATTCCCTTCAGGTAAGGCA
Pdgfrb forward<br>Pdgfrb reverse	TTCCAGGAGTGATACCAGCTT<br>AGGGGGCGTGATGACTAGG
Scx forward<br>Scx reverse	CTGGCCTCCAGCTACATTTCT<br>GTCACGGTCTTTGCTCAACTT
Tnmd forward<br>Tnmd reverse	ACACTTCTGGCCCGAGGTAT<br>GACTTCCAATGTTTCATCAGTGC
TnC forward<br>TnC reverse	ACGGCTACCACAGAAGCTG<br>ATGGCTGTTGTTGCTATGGCA
Pax7 forward<br>Pax7 reverse	GCTACCAGTACAGCCAGTATG<br>GTCACTAAGCATGGGTAGATG
Gapdh forward<br>Gapdh reverse	AGGTCGGTGTGAACGGATTTG<br>TGTAGACCATGTAGTTGAGGTCA

Supplementary information, Table 3: List of primers used for RT-qPCR analyses.

Bibliography

1. Chan, C. K. F. *et al.* Identification and specification of the mouse skeletal stem cell. *Cell* **160**, 285–298 (2015).

2. Debnath, S. *et al.* Discovery of a periosteal stem cell mediating intramembranous bone formation. *Nature* **562**, 133–139 (2018).

3. Zhou, B. O., Yue, R., Murphy, M. M., Peyer, J. G. & Morrison, S. J. Leptin-receptor-expressing mesenchymal stromal cells represent the main source of bone formed by adult bone marrow. *Cell Stem Cell* **15**, 154–168 (2014).

4. Park, D. *et al.* Endogenous bone marrow MSCs are dynamic, fate-restricted participants in bone maintenance and regeneration. *Cell Stem Cell* **10**, 259–272 (2012).

5. Greenbaum, A. *et al.* CXCL12 in early mesenchymal progenitors is required for haematopoietic stem-cell maintenance. *Nature* **495**, 227–230 (2013).

6. Méndez-Ferrer, S. *et al.* Mesenchymal and haematopoietic stem cells form a unique bone marrow niche. *Nature* **466**, 829–834 (2010).

7. Worthley, D. L. *et al.* Gremlin 1 identifies a skeletal stem cell with bone, cartilage, and reticular stromal potential. *Cell* **160**, 269–284 (2015).

8. Schaum, N. *et al.* Single-cell transcriptomics of 20 mouse organs creates a Tabula Muris. *Nature* **562**, 367–372 (2018).

9. Giordani, L. *et al.* High-Dimensional Single-Cell Cartography Reveals Novel Skeletal Muscle-Resident Cell Populations. *Mol. Cell* **74**, 609-621.e6 (2019).

10. Scott, R. W., Arostegui, M., Schweitzer, R., Rossi, F. M. V. & Underhill, T. M. Hic1 Defines Quiescent Mesenchymal Progenitor Subpopulations with Distinct Functions and Fates in Skeletal Muscle Regeneration. *Cell Stem Cell* **25**, 797-813.e9 (2019).

11. Ionescu, A. M. *et al.* PTHrP Modulates Chondrocyte Differentiation through AP-1 and CREB Signaling. *J. Biol. Chem.* **276**, 11639–11647 (2001).

12. Tufan, A. C., Daumer, K. M., DeLise, A. M. & Tuan, R. S. Ap-1 transcription factor complex is a target of signals from both Wnt-7a and N-cadherin-dependent cell-cell adhesion complex during the regulation of limb mesenchymal chondrogenesis. *Exp. Cell Res.* **273**, 197–203 (2002).

13. Vale-Cruz, D. S., Ma, Q., Syme, J. & LuValle, P. A. Activating transcription factor-2 affects skeletal growth by modulating pRb gene expression. *Mech. Dev.* **125**, 843–856 (2008).

14. Ionescu, A. M. *et al.* ATF-2 cooperates with Smad3 to mediate TGF-β effects on chondrocyte maturation. *Exp. Cell Res.* **288**, 198–207 (2003).

15. Beier, F., Lee, R. J., Taylor, A. C., Pestell, R. G. & Luvalle, P. Identification of the cyclin D1 gene as a target of activating transcription factor 2 in chondrocytes. *Proc. Natl. Acad. Sci. U. S. A.* **96**, 1433–1438 (1999).

16. Fisher, M. C., Clinton, G. M., Maihle, N. J. & Dealy, C. N. Requirement for ErbB2/ErbB signaling in developing cartilage and bone. *Dev. Growth Differ.* **49**, 503–513 (2007).

17. Jia, H. *et al.* EGFR signaling is critical for maintaining the superficial layer of articular cartilage and preventing osteoarthritis initiation. *Proc. Natl. Acad. Sci. U. S. A.* **113**, 14360–14365 (2016).

18. Lui, J. C. *et al.* Cartilage-Targeted IGF-1 Treatment to Promote Longitudinal Bone Growth. *Mol. Ther.* **27**, 673–680 (2019).

19. Lui, J. C. *et al.* EZH1 and EZH2 promote skeletal growth by repressing inhibitors of chondrocyte proliferation and hypertrophy. *Nat. Commun.* **7**, (2016).

20. Fischer, J., Knoch, N., Sims, T., Rosshirt, N. & Richter, W. Time-dependent contribution of BMP, FGF, IGF, and HH signaling to the proliferation of mesenchymal stroma cells during chondrogenesis. *J. Cell. Physiol.* **233**, 8962–8970 (2018).

21. Zhang, M. *et al.* Smad3 prevents β-catenin degradation and facilitates β-catenin nuclear translocation in chondrocytes. *J. Biol. Chem.* **285**, 8703–8710 (2010).

22. Fang, M., Alfieri, C. M., Hulin, A., Conway, S. J. & Yutzey, K. E. Loss of β-catenin promotes chondrogenic differentiation of aortic valve interstitial cells. *Arterioscler. Thromb. Vasc. Biol.* **34**, 2601–2608 (2014).

23. Usami, Y. *et al.* Possible Contribution of Wnt-Responsive Chondroprogenitors to the Postnatal Murine Growth Plate. *J. Bone Miner. Res.* **34**, 964–974 (2019).

24. Kosaka, T. *et al.* RAGE, receptor of advanced glycation endoproducts, negatively regulates chondrocytes differentiation. *PLoS One* **9**, (2014).

25. Zhang, H. *et al.* Intramembranous ossification and endochondral ossification are impaired differently between glucocorticoid-induced osteoporosis and estrogen deficiency-induced osteoporosis. *Sci. Rep.* **8**, (2018).

26. Hara, E. S. *et al.* Fluocinolone acetonide is a potent synergistic factor of TGF-β3-associated chondrogenesis of bone marrow-derived mesenchymal stem cells for articular surface regeneration. *J. Bone Miner. Res.* **30**, 1585–1596 (2015).

27. Diederichs, S. *et al.* Regulation of WNT5A and WNT11 during MSC in vitro chondrogenesis: WNT inhibition lowers BMP and hedgehog activity, and reduces hypertrophy. *Cell. Mol. Life Sci.* **76**, 3875–3889 (2019).

28. Wang, X., Cornelis, F. M. F., Lories, R. J. & Monteagudo, S. Exostosin-1 enhances canonical Wnt signaling activity during chondrogenic differentiation. *Osteoarthr. Cartil.* **27**, 1702–1710 (2019).
