# Supplementary material for: Direct contribution of skeletal muscle mesenchymal progenitors to bone repair
Source: Nat Commun. 2021 May 17;12:2860. doi: 10.1038/s41467-021-22842-5 (PMC8128920; doi:10.1038/s41467-021-22842-5)
Supplement: Supplementary file 3 — Reporting Summary [file 41467_2021_22842_MOESM3_ESM.pdf]

nature research

Corresponding author(s): Colnot Celine

Last updated by author(s): 2021/02/21

Reporting Summary

Nature Research wishes to improve the reproducibility of the work that we publish. This form provides structure for consistency and transparency in reporting. For further information on Nature Research policies, see our Editorial Policies and the Editorial Policy Checklist.

Statistics

For all statistical analyses, confirm that the following items are present in the figure legend, table legend, main text, or Methods section.

n/a	Confirmed	
☐	☒	The exact sample size (*n*) for each experimental group/condition, given as a discrete number and unit of measurement
☐	☒	A statement on whether measurements were taken from distinct samples or whether the same sample was measured repeatedly
☐	☒	The statistical test(s) used AND whether they are one- or two-sided<br>*Only common tests should be described solely by name; describe more complex techniques in the Methods section.*
☐	☒	A description of all covariates tested
☐	☒	A description of any assumptions or corrections, such as tests of normality and adjustment for multiple comparisons
☐	☒	A full description of the statistical parameters including central tendency (e.g. means) or other basic estimates (e.g. regression coefficient) AND variation (e.g. standard deviation) or associated estimates of uncertainty (e.g. confidence intervals)
☐	☒	For null hypothesis testing, the test statistic (e.g. *F*, *t*, *r*) with confidence intervals, effect sizes, degrees of freedom and *P* value noted<br>*Give P values as exact values whenever suitable.*
☒	☐	For Bayesian analysis, information on the choice of priors and Markov chain Monte Carlo settings
☒	☐	For hierarchical and complex designs, identification of the appropriate level for tests and full reporting of outcomes
☒	☐	Estimates of effect sizes (e.g. Cohen's *d*, Pearson's *r*), indicating how they were calculated

Our web collection on statistics for biologists contains articles on many of the points above.

Software and code

Policy information about availability of computer code

Data collection	FACS data was collected with FACS Fortessa X-20.<br>Cells were sorted on BS FACS Aria II SORP using BD FACS Diva Software Version v8.<br>Pictures were acquired on Zeiss Imager D1 AX10 light microscope (Zeiss), Spinning Disk (Zeiss) or LSM700 confocal microscope (Zeiss).<br>qPCR data were collected with 7300 Real-Time PCR System version v1.3.1.<br>Single cell data were generated with 10x Chromium technology. RNA were sequenced by NovaSeq 600 sequencing machine.<br>X-ray microCT data were acquired on Quantum FXCaliper from Life Technologies.
Data analysis	GraphPad PRISM software Version 6.0a was used for statistical analyses<br>FACS data were analysed using FlowJo software v10.5.3<br>Histomorphometric data were measured using ZEN v1.1.2.0 software.<br>Confocal images were processed using ImageJ v2.1.0.<br>Cell Ranger v5.0.1 was used to demultiplex and align single cell data<br>RStudio v1.2.1335 and Seurat v3.0.2 were used to analyze Single cell RNAseq data<br>Monocle3 v0.2.3.0 was used to performed pseudotime analysis<br>qPCR data were analyzed using Excel v14.7.3 (170325)<br>X-ray microCT analyses were performed using DataViewer64 software v1.5.2.4 and CTan software v1.17.7.2.

For manuscripts utilizing custom algorithms or software that are central to the research but not yet described in published literature, software must be made available to editors and reviewers. We strongly encourage code deposition in a community repository (e.g. GitHub). See the Nature Research guidelines for submitting code & software for further information.

Data

Policy information about availability of data

All manuscripts must include a data availability statement. This statement should provide the following information, where applicable:

- Accession codes, unique identifiers, or web links for publicly available datasets
- A list of figures that have associated raw data
- A description of any restrictions on data availability

GSE164573, GSE110878, GSE110038

Field-specific reporting

Please select the one below that is the best fit for your research. If you are not sure, read the appropriate sections before making your selection.

[x] Life sciences [ ] Behavioural & social sciences [ ] Ecological, evolutionary & environmental sciences

For a reference copy of the document with all sections, see nature.com/documents/nr-reporting-summary-flat.pdf

Life sciences study design

All studies must disclose on these points even when the disclosure is negative.

Sample size	Based on our previous work (Duchamp de Lageneste O. et al, Periosteum contains skeletal stem cells with high bone regenerative potential controlled by Periostin, Nature Communications, 2018 Feb 22;9(1):773, Julien A. et al, FGFR3 in Periosteal Cells Drives Cartilage-to-Bone Transformation in Bone Repair, Stem Cell Reports, 2020 Oct 13;15(4):955-967), groups of 3 to 6 animals is sufficient for quantitative analyses.
Data exclusions	All samples were included except for fractures that were proximal and/or distal comminuted fractures. Exclusion criteria were pre-established knowing that proximal/distal comminuted fractures exhibit changes in fracture healing parameters as volume of callus, cartilage and bone, independently of other experimental parameters.
Replication	For each experimental group, mice were from different litters and samples obtained from multiple experiments (at least 2) to generate biological replicates. For RT-qPCR analyses, results are reported for 3 to 4 biological replicates, and 2 to 3 technical replicates were generated. All technical replicates were successful.
Randomization	No specific randomization methods were used for the study. However, experimental groups were homogeneous and composed of equivalent animals based on gender, age and genotype.
Blinding	All analyses were performed using a blind numbering system. Unique identifier number was assigned to each mouse, and samples were collected, processed and analyzed using this blinding numbering system.

Reporting for specific materials, systems and methods

We require information from authors about some types of materials, experimental systems and methods used in many studies. Here, indicate whether each material, system or method listed is relevant to your study. If you are not sure if a list item applies to your research, read the appropriate section before selecting a response.

Materials & experimental systems

n/a	Involved in the study	
[ ]	[x]	Antibodies
[x]	[ ]	Eukaryotic cell lines
[x]	[ ]	Palaeontology and archaeology
[ ]	[x]	Animals and other organisms
[x]	[ ]	Human research participants
[x]	[ ]	Clinical data
[x]	[ ]	Dual use research of concern

Methods

n/a	Involved in the study	
[x]	[ ]	ChIP-seq
[ ]	[x]	Flow cytometry
[x]	[ ]	MRI-based neuroimaging

Antibodies

Antibodies used	Antibodies for FACS were all diluted at 1:200 (supplier, catalog number, clone)<br>CD31-PECy7 (BD Biosciences, catalog number 561410, clone 309)<br>CD45-PECy7 (BD Biosciences, catalog number 552848, clone 30-F11)<br>CD11b-PECy7 (BD Biosciences, catalog number 552850, clone M1/70)<br>CD34-AF700 (BD Biosciences, catalog number 560518, clone RAM34)<br>Sca1-BV650 (BD Biosciences, catalog number 740450, clone D7)

CD29-APC (Miltenyi Biotec, catalog number 130-102-557, clone HMβ1-1)
PDGFRβ-APC (Invitrogen, catalog number 17-1402-80, clone APB5)
PDGFRα-BV711 (BD Biosciences, catalog number 740740, clone APA5)

Primary antibodies used for immunofluorescence (dilution, catalog number, supplier)
goat anti-mouse periostin (1/400, catalog number AF2955, R&D)
goat anti-mouse PDGFRα (1/200, catalog number AF1062, R&D)
rabbit anti-mouse NG2 (1/50, catalog number AB5320, Merck)
rabbit anti-mouse PDGFRβ (1/200, catalog number ab32570, Abcam)
goat anti-mouse CD29 (1/50, catalog number 026202, R&D)
anti-mouse αSMA-Cy5 (1/200, catalog number AC12-0159-11, clone 1A4, Clinisciences).
rabbit anti-mouse ColX (1/200, catalog number ab58632 Abcam)
rabbit anti-mouse Osx (1/400, catalog number ab22552 Abcam)
rabbit anti-mouse S100b (1/200, catalog number Z0310, Agilent)

Secondary antibodies used for immunofluorescence: goat anti-rabbit AF647 (1/250, catalog number 21245, Life Technologies), donkey anti-goat AF647 (1/500, catalog number ab150135, Abcam), goat anti-rabbit AF546 (1/250, catalog number A11010, Invitrogen) and donkey anti-goat AF488 antibody (1/250, catalog number A11055, Invitrogen)

Validation

All FACS antibodies were validated using UltraCompBeads (REF 01-2222-42, Thermo Fischer). FMO controls were used to determine background level of each color and separate positive and negative signal.
Antibody/Fluorochrome Supplier Validation)
CD31-PECy7/ http://www.bdbiosciences.com/eu/applications/research/stem-cell-research/cancer-research/mouse/pe-cy7-rat-anti-mouse-cd31-390/p/561410
CD45-PECy7/ http://www.bdbiosciences.com/eu/applications/research/stem-cell-research/cancer-research/mouse/pe-cy7-rat-anti-mouse-cd45-30-f11/p/552848
CD11b-PECy7 / http://www.bdbiosciences.com/us/applications/research/stem-cell-research/mesenchymal-stem-cell-markers-bone-marrow/mouse/negative-markers/pe-cy7-rat-anti-cd11b-m170/p/552850
CD34-AF700 / http://www.bdbiosciences.com/us/applications/research/stem-cell-research/cancer-research/mouse/alexa-fluor-700-rat-anti-mouse-cd34-ram34/p/560518
Sca1-BV650 / https://www.bdbiosciences.com/us/reagents/research/antibodies-buffers/immunology-reagents/anti-mouse-antibodies/cell-surface-antigens/bv650-rat-anti-mouse-ly-6ae-d7/p/740450
CD29-APC / https://www.miltenyibiotec.com/FR-en/products/macs-flow-cytometry/antibodies/primary-antibodies/cd29-antibodies-mouse-hmb1-1-1-10.html#apc:30-ug-in-1-ml
PDGFRβ-APC / https://www.thermofisher.com/antibody/product/CD140b-PDGFRB-Antibody-clone-APB5-Monoclonal/17-1402-82

All antibodies used for immunofluorescence staining were validated by manufacturers or in published studies:
goat anti-mouse periostin (1/400, catalog number AF2955, R&D): Duchamp de Lageneste O. et al, Periosteum contains skeletal stem cells with high bone regenerative potential controlled by Periostin, Nature Communications, 2018 Feb 22;9(1):773

goat anti-mouse PDGFRα (1/200, catalog number AF1062, R&D): Uezemi A. et al, Mesenchymal progenitors distinct from satellite cells contribute to ectopic fat cell formation in skeletal muscle, Nature Cell Biology, 2010 Feb;12(2):143-52.

rabbit anti-mouse NG2 (1/50, catalog number AB5320, Merck): Koatallari E. et al, Pericytes in the myovascular niche promote post-natal myofiber growth and satellite cell quiescence, Development. 2015 Apr 1;142(7):1242-53.

rabbit anti-mouse PDGFRβ (1/200, catalog number ab32570, Abcam): Abcam cite NIH/3T3 cells as positive control. https://www.abcam.com/pdgfr-beta-antibody-y92-c-terminal-ab32570.html

goat anti-mouse CD29 (1/50, catalog number 026202, R&D): Duchamp de Lageneste O. et al, Periosteum contains skeletal stem cells with high bone regenerative potential controlled by Periostin, Nature Communications, 2018 Feb 22;9(1):773

anti-mouse αSMA-Cy5 (1/200, catalog number AC12-0159-11, clone 1A4, Clinisciences): https://abcore.com/anti-alpha-smooth-muscle-actin-antibody-cy55-p-ac12-0159-11/

rabbit anti-mouse ColX (1/200, catalog number ab58632 Abcam): Julien A. et al, FGFR3 in Periosteal Cells Drives Cartilage-to-Bone Transformation in Bone Repair, Stem Cell Reports, 2020 Oct 13;15(4):955-967.

rabbit anti-mouse Osx (1/400, catalog number ab22552 Abcam): Julien A. et al, FGFR3 in Periosteal Cells Drives Cartilage-to-Bone Transformation in Bone Repair, Stem Cell Reports, 2020 Oct 13;15(4):955-967.

rabbit anti-mouse S100b (1/200, catalog number Z0310, Agilent): Radomska K.J. et al, Cellular Origin, Tumor Progression, and Pathogenic Mechanisms of Cutaneous Neurofibromas Revealed by Mice with Nf1 Knockout in Boundary Cap Cells, Cancer Discovery, 2019 Jan;9(1):130-147.

Secondary antibodies were incubated without primary antibody as control.

Animals and other organisms

Policy information about studies involving animals; ARRIVE guidelines recommended for reporting animal research

Laboratory animals	C57BL/6ScNj, beta-actinGFP, Prx1Cre, Pax7CreERT2, Rosa-tdTomato-EGFP (RosamTmG) and RosaYFP mice were obtained from Jackson Laboratory (Bar Harbor, ME) and maintained on a C57BL6/J background. Animals used for all experiments were males or females 10 to 14-week-old and experimental groups were homogeneous in terms of animal gender and age.
Wild animals	No wild type animals were used
Field-collected samples	No of filed-collected samples were used
Ethics oversight	All procedures were approved by the Paris Est Créteil and Paris Universities Ethical Committees (APAFIS#19295-2019052015468705 v1).

Note that full information on the approval of the study protocol must also be provided in the manuscript.

Flow Cytometry

Plots

Confirm that:

- [x] The axis labels state the marker and fluorochrome used (e.g. CD4-FITC).
- [x] The axis scales are clearly visible. Include numbers along axes only for bottom left plot of group (a 'group' is an analysis of identical markers).
- [x] All plots are contour plots with outliers or pseudocolor plots.
- [ ] A numerical value for number of cells or percentage (with statistics) is provided.

Methodology

Sample preparation	Prx1Cre;RosamTmG mice were sacrificed by cervical dislocation. Skin and fascia were removed. TA, EDL, plantaris, gastrocnemius and soleus muscles surrounding the tibia were dissected from tendon to tendon avoiding periosteum or bone marrow cell contamination. In a petri dish with 1mL of DMEM medium (21063029, Invitrogen), tendon and fat were removed, and skeletal muscles were minced using scissors. Skeletal muscles were transferred in 3mL of digesting medium containing DMEM (21063029, Invitrogen), 1% Trypsin (210234, Roche), 1% collagenase D (11088866001, Roche) and incubated in a water bath at 37°C for 2 hours. Every 20 min individualized cells were removed and transferred into ice-cold growth medium containing αMEM (32561029, Life Technologies) with 1% penicillin-streptomycin (P/S, 15140122, Life Technologies), 20% lot-selected non-heat-inactivated fetal bovine serum (FBS, 10270106, Life Technologies) and 10ng/ml bFGF (3139-FB-025/CF, R&D) and fresh digesting medium was added to the undigested tissue. This step was repeated until all skeletal muscle was digested. Cells were then filtered through 100µm filters (352360, Dutscher) and 40µm filters (352340, Dutscher), centrifuged 10min at 1500 rpm and resuspended in 3mL of growth medium.<br>For cell sorting, skeletal muscle cells were resuspended in 1mL of sorting medium containing DMEM medium (21063029, Invitrogen) with 2% of FBS and 1% of P/S. Cell viability marker Sytox blue (1/1000, S34857, Thermofischer) was added just before sorting. Cell sorting was performed on BD FACS Aria II SORP (BD Biosciences) and cells were collected in growth medium. For cell transplantation, 150 000 freshly sorted skeletal muscle cells were embedded in TissuCol® kit TISSEEL (human fibrogen 15mg/mL and thrombin 9mg/mL, Baxter, France) according to manufacturer's instructions. Open fracture was performed as described above and cell pellets were implanted at the fracture site.
Instrument	FACS data were collected with FACS Fortessa X-20.
Software	FACS data were collected with BD FACS Diva Software v8 and analysed using FlowJo software v10.5.3.
Cell population abundance	For sorted populations purity was determined by re-analysis for the target population immediately post sorting based on cell surface markers. Purity was >99% for each target population.
Gating strategy	Beads (REF 01-2222-42, Thermo Fischer) were used for initial compensation set up and FMO controls were used to determine background level of each colour and positive vs negative signal. We selected cells excluding debris, doublet and dead cells (negative for SytoxBlue). Gating strategy is available in Supplementary Figure 4a-b.

- [x] Tick this box to confirm that a figure exemplifying the gating strategy is provided in the Supplementary Information.
